# Refocusing Neuroprotection in Cerebral Reperfusion Era: New Challenges and Strategies

**DOI:** 10.3389/fneur.2018.00249

**Published:** 2018-04-23

**Authors:** Xiao-Yi Xiong, Liang Liu, Qing-Wu Yang

**Affiliations:** Department of Neurology, Xinqiao Hospital, The Army Medical University (Third Military Medical University), Chongqing, China

**Keywords:** acute ischemic stroke, collateral circulation, endovascular therapy, hemorrhagic transformation, neuroprotection

## Abstract

Pathophysiological processes of stroke have revealed that the damaged brain should be considered as an integral structure to be protected. However, promising neuroprotective drugs have failed when translated to clinical trials. In this review, we evaluated previous studies of neuroprotection and found that unsound patient selection and evaluation methods, single-target treatments, etc., without cerebral revascularization may be major reasons of failed neuroprotective strategies. Fortunately, this may be reversed by recent advances that provide increased revascularization with increased availability of endovascular procedures. However, the current improved effects of endovascular therapy are not able to match to the higher rate of revascularization, which may be ascribed to cerebral ischemia/reperfusion injury and lacking of neuroprotection. Accordingly, we suggest various research strategies to improve the lower therapeutic efficacy for ischemic stroke treatment: (1) multitarget neuroprotectant combinative therapy (cocktail therapy) should be investigated and performed based on revascularization; (2) and more efforts should be dedicated to shifting research emphasis to establish recirculation, increasing functional collateral circulation and elucidating brain–blood barrier damage mechanisms to reduce hemorrhagic transformation. Therefore, we propose that a comprehensive neuroprotective strategy before and after the endovascular treatment may speed progress toward improving neuroprotection after stroke to protect against brain injury.

## Introduction

During the past two decades, significant global and regional burdens have been attributed to stroke, which are still increasing (1). Although good preventive measures have been taken to decrease the age-standardized incidence of stroke (1), the insufficient clinical therapies for patients with acute stroke have driven researchers to explore more promising therapeutic strategies that can be translated from bench to bedside with the goal of an ideal cure.

Neuronal injury in stroke has gradually been recognized to result from a surge in the activation of complicated pathophysiological pathways, from ischemic damage initiation to secondary brain injury; all these advantageous and disadvantageous events are interlinked (2, 3). Within a conspicuous time frame, excitotoxicity, oxidative and nitrosative stress, and inflammatory mechanisms predominate at the core of ischemic brain damage (2, 4) and have prompted researchers to develop intervention measures to improve the neurofunctions destroyed by stroke in patients. However, more than 1,000 promising preclinical therapeutic drugs that have aimed to salvage ischemic brain injury have yielded disappointing results in clinical trials (5). Numerous reasons have been recently discussed and explained in many articles and symposia, such as questioning the non-comparable stroke models in rodents with clinical patients and criticizing the design of clinical neuroprotection trials (6–8). Accordingly, the Stroke Therapy Academic Industry Roundtable (STAIR) recommendations and some guidelines have been proposed to resolve these issues and have greatly improved the methodology of stroke trials. However, several years have passed, and there are still no effective therapeutic strategies that have been shown to improve the outcomes of stroke, which has caused stroke neurologists to reconsider the reasons behind the failure of the clinical trials.

Ischemic stroke is a vascular and neural disease that is caused by the deprivation of blood supply when arteries are occluded. Therefore, successful recanalization of the occluded arteries would deliver more neuroprotectants to salvageable brain tissues. Fortunately, endovascular treatment has been the greatest advancement in stroke therapy in the past two decades and now is the new standard treatment for patients with acute ischemic stroke (9). This approach has created great opportunities for better estimating the effectiveness of neuroprotectants *via* improved recanalization that might be accomplished using this endovascular therapy (4, 9). However, in addition to the beneficiary effects, the recanalization of the occluded arteries could also result in harmful effect after ischemic stroke, for example, the cerebral ischemia/reperfusion injuries (e.g., hemorrhagic transformation) (10).

This review aims to draw on lessons from the history of neuroprotection in acute ischemic stroke to summarize the main reasons behind the failed translation of neuroprotection from bench to bedside in acute ischemic stroke patient and to provide some promising approaches for acute ischemic stroke therapy based on recent novel strategies of completed randomized controlled trials (RCTs) in patients with acute ischemic stroke. Given the limitations (e.g., cerebral ischemia/reperfusion injuries) of this current promising endovascular treatment, this review also focuses on future directions for investigating the mechanisms underlying hemorrhagic transformation and increasing functional collateral circulation and recirculation.

## Neuroprotection History

Neuroprotection has received significant attention over the past 30 years. For acute ischemic stroke, neuroprotection can be defined as strategies, applied alone or in combination, that directly or indirectly target the brain parenchyma with the aim of antagonizing the harmful molecular and cellular events caused by ischemia, allowing brain cells to survive and the penumbra to be spared (6, 11).

Neuroprotection studies gradually emerged from the 1970s to 1990s and developed during the 2000s. From the 1990s, with the goal of identifying strategies to reduce neuronal injury, researchers began to focus on the underlying mechanisms of ischemic brain injury. The rapidly occurring excitotoxicity that resulted from energy failure caused by the disturbance of blood supply was intensely studied and identified as the first molecular mechanism of ischemic brain tissue damage. Therefore, the reduction of this type of neuronal death contributed to an understanding of many underlying mechanisms and relevant therapeutic targets for the treatment of acute ischemic stroke (3). Excitatory amino acids, *N*-methyl-d-aspartate receptor signaling, and calcium channels have been shown to accelerate neuronal cell death (6). Unfortunately, almost all promising agents targeting these were ineffective in clinical trials (5).

**Figure 1:**
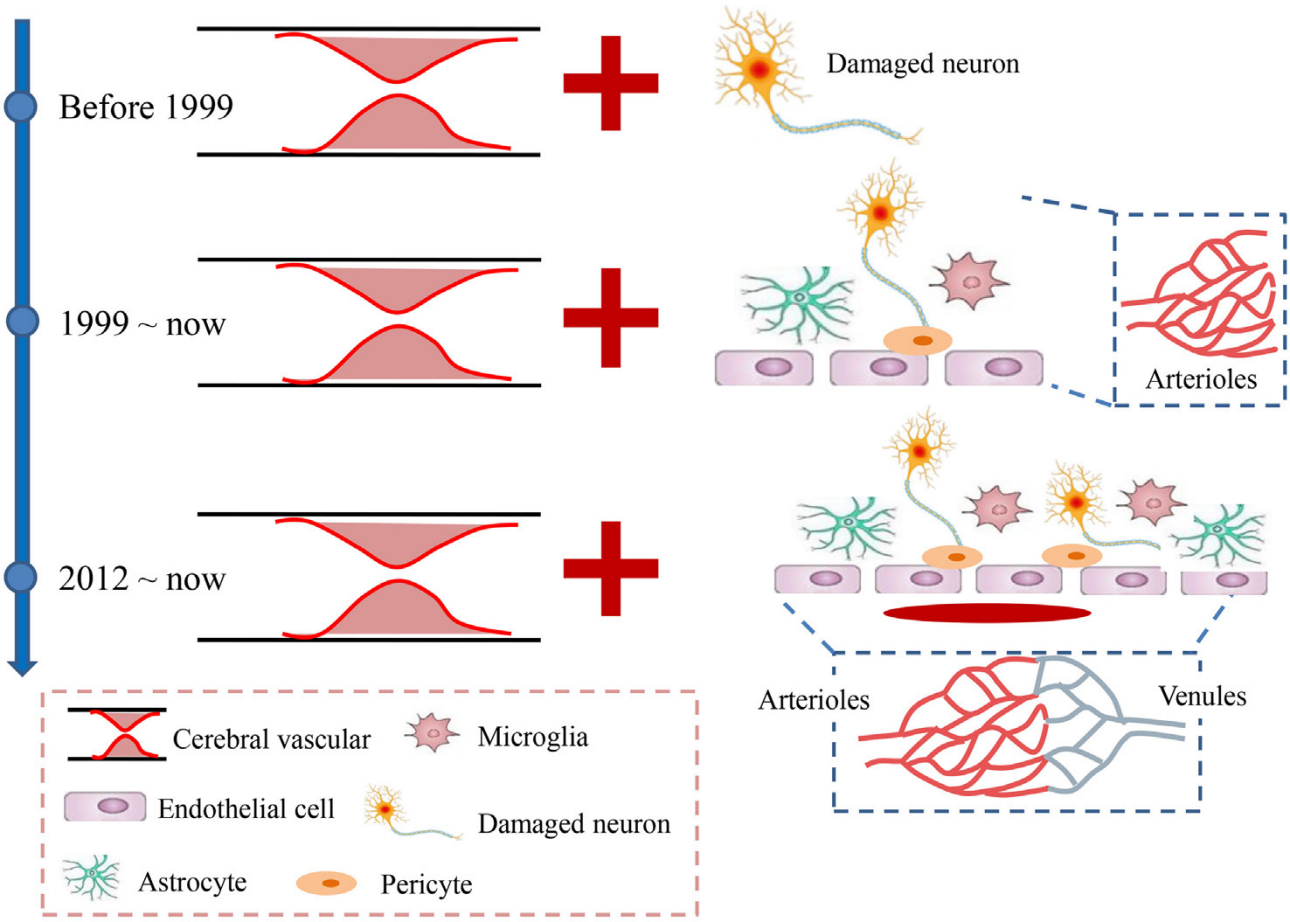
Developmental history of acute ischemic stroke. Before 1999, stroke was mainly considered a vascular disease caused by the occlusion of cerebral arteries, which led to neuronal injury. Since 1999, the neurovascular unit, which includes the neuron, microglia, astrocytes, and endothelial cells, was introduced to suggest that all the levels involved the ischemic brain damage should be considered as integrated. In 2012, the neurovascular unit was expanded to a vascular neural network, which emphasized more attention on the venous system and suggested maintaining recirculation after stroke.

Apart from the rapid excitotoxicity, growing concerns about the subsequent increase in oxidative injury and central and peripheral inflammation that are involved in many aspects of cellular and molecular events further support the idea that ischemic stroke is not only a vascular disease, as many neural and vascular cells (e.g., microglia/macrophages, astrocytes, neurons, and endothelial cells) are involved in these cascades. This prompted the concept of a neurovascular unit defined as a triad consisting of endothelial cells, astrocytes, and neurons (12); these cells are considered to be a unique functional entity due to the complexity of interactions among the perivascular cell types (12–15) (Figure 1). The cells easily and rapidly communicate with each other due to the high specificity enabled by the submillimeter scale of the unit (16, 17). However, the micrometer scale of neurovascular unit would largely exclude the contributions of vascular cells to the pathophysiology of brain injury (12). Therefore, the concept of a vascular neural network was proposed to encompass and combine the original concept of the neurovascular unit with arterial smooth muscle cells, endothelial cells, and perivascular nerves in cerebrovascular physiology and pathology (12). This new concept expanded the physical components of the neurovascular unit to even include the venous system (Figure 1), which was also considered to be involved in the maintenance of normal brain functions (18). Both concepts emphasized that the brain injury caused by stroke should be considered as highly important for protection, and therapies should not exclusively focus on neuronal targeting. However, although the concept of the neurovascular unit was proposed in 2002, still no neuroprotectants targeting on the neurovascular unit or network have been used for clinical patients with acute stroke. For instance, disodium 2,4-disulphophenyl-*N*-tert-butylnitrone (NXY-059), a novel nitrone with free radical (can affect all cells of the neurovascular unit) trapping properties (19, 20), reduces infarct volume and motor impairment in experimental models of stroke in rodents (21, 22), rabbits (23), and primates (24, 25). However, when translated into clinical trials, the neuroprotective effects of NXY-059 were negative (26–28). In addition, edaravone, a novel free radical scavenger, has induced both potential neuroprotective effects by ameliorating neuronal damage and improved functional outcomes in ischemic stroke animal models (29, 30) and some clinical trials (31). Accordingly, edaravone has already been clinically used in some Asian countries (such as Japan and China) due to its potential neuroprotective effects although it still does not have marketing authorization in Europe or America.

In addition, roles of innate immune responses in the brain injury caused by ischemic stroke have received significant attention in recent years (2). For example, the peripheral innate immune cells infiltrated into brain would also aggravate or alleviate the brain injury caused ischemic stroke (32, 33). Therefore, targeting some of these innate immune responses (both central and peripheral) has led to significant improvements in neurofunction in animal stroke models (34–36) and some pilot clinical trials (37, 38). These results strongly suggest that acute ischemic stroke is not just a disease that occurs in the brain but may also be a systemic disease influenced by the peripheral innate immune cells and some other known and unknown factors. Although these pilot clinical trials have shown promising neuroprotection against ischemic stroke, further large-scale, multicentre clinical trials are needed to re-evaluate the neuroprotective effects.

Furthermore, various drugs with promising multifaceted therapeutic targets have been demonstrated to play positive neuroprotective effects in animal stroke models. For instance, the DL-3-n-butylphthalide (NBP) may act by improving mitochondria function and energy metabolism (39), decreasing oxidative damage and apoptosis (40), reducing inflammatory responses (41), and enhancing regional blood flow and angiogenesis (42) to protect against ischemic brain damage and to result in reduction of infarct volume (39, 43). Clinical trials have also shown that NBP can improve outcomes of patients with stroke (44, 45). In addition to NBP, statins (46, 47), citicoline (48), and stem cells (49) are also purported to have multiple mechanisms of action for acute ischemic stroke. Besides, some smart delivery methods, including TAT protein transduction, nasal delivery of peptides, etc., might be effective neuroprotective therapies for acute ischemic stroke, because they also have pleiotropic actions and do not depend extensively on reflow of blood flow (50–52). While, large randomized, double-blind, placebo-controlled studies are needed to assess the safety and efficacy of these multifaceted therapeutics in patients with acute ischemic stroke.

Although neuroprotective research has come to a difficult bottleneck, recent developments in endovascular treatment technologies may help to solve this predicament. The higher rates of revascularization provided by endovascular therapy can timely deliver blood to salvageable tissues without increasing symptomatic intracranial hemorrhage within 90 days compared to control groups (53). Therefore, endovascular treatment is now regarded as the new standard for patients with acute ischemic stroke (9, 54, 55).

Thus far, neuroprotection is still a promising option for acute ischemic stroke treatment although clinical trials have repeatedly failed. To solve this issue, many suggestions and guidelines have been proposed to improve outcomes. For example, the STAIR committee has suggested that non-human primates (NHPs) should be used for preclinical, translational stroke studies to address potential discrepancies between animals and human studies (56). Preclinical multicentre studies are also suggested to improve the translation of treatment efficacy from bench to bedside. However, although some studies included many of the STAIR guidelines, they still failed to improve the outcomes of patients. For example, the neuroprotective effects of NXY-059 investigated by Green (57) were developed in accordance with the criteria proposed by the STAIR (56) yet still failed to achieve the expected efficacy (58). These unexpected findings have urged scientists and clinicians to rethink and re-analyze the path toward stroke drug discovery.

## Reasons for Failure of Neuroprotection

Elucidating the mechanisms at the cellular and molecular levels has led to the identification of many promising targets for neuroprotection. More than 1,000 potential neuroprotective agents have been announced with greater neuroprotection in stroke models; however, only 114 of these have been translated to the clinic, of which only the alteplase has been shown to improve patient outcome (5). The first STAIR recommendations were published in 1999 to promote increased rates of successful clinical translation. Since then, although some promising agents have been confirmed in stroke models of NHPs, which more closely resemble the physiology of humans, before undergoing clinical trials, ultimate proof of successful translation from bench to bedside in stroke research is still lacking. These continuous failures in clinical translation have led to pessimism regarding the possibility of obtaining good outcomes of neuroprotection after stroke. It is important to determine why neuroprotection has not been achieved with more than 1,000 potential drugs, while in the other research fields, such as cancer, several antitumor agents are already being used in clinical practice. Therefore, instead of abandoning neuroprotection as a strategy, it is important to rethink and re-evaluate the lessons learned from neuroprotection research and to focus on how we can better determine the gaps between animal and clinical studies.

### Mismatch Between Animals and Humans

It is undeniable that most of stroke animal models have been young rodents aged less than 3 months, which have healthier and better stress resistance abilities than older rodents. In addition, many comorbidities exist in stroke patients. Therefore, the pathophysiological baseline of young rodents in stroke research is largely mismatched with stroke patients. Thus, preclinical randomized controlled multicentre trials (pRCTs), like the anti-CD49d treatment for acute brain ischemia investigated by a pRCT (59), have been suggested to help bridge the gap between experimental laboratory research and clinical trials (60–62). Because the preclinical studies would judge the conclusion that a drug “was successful” in animals, for example, an individual animal meta-analysis showed that NXY-059 was neuroprotective in experimental stroke although bias may have resulted in efficacy being overestimated (63). Moreover, the use of NHPs, especially those with a high degree of anatomic resemblance to the human brain, vascular supply, and collateral circulation in brain regions, as a major stroke model for preclinical trials to develop strategies may also be benefited for the clinical transformation studies (64). Besides, there are some aspects that may also help us to realize and solve the failures, for example, (1) long-term outcome measures in animal models is required while not just only short-term evaluations; (2) behavioral tests for animal models differ in different researches; (3) longitudinal imaging in individual mice about the structural and functional plasticity of vascular neural networks should be added to provide complementary evidence toward efficacy; and (4) lacking of effective quality control system for animal researches, like statistical guidelines, ARRIVE guidelines, registration of preclinical study designs, and outcome evaluations, etc. Although many articles and symposia have discussed this topic and have provided guidelines and suggestions to resolve this issue, it remains unknown how these mismatches and shortages influence the translational effects of stroke research.

### Limitations of Current Clinical Trial Strategies

Almost all clinical trials have used the National Institute of Health stroke scale (NIHSS) scores as a major inclusion criterion for patients with acute ischemic stroke. Generally, the NIHSS score ranges from 0 to 42 (with higher scores denoting greater disability): a score of 0 suggests a normal neurological examination, 1 suggests negligible abnormality (65), and an NIHSS score of ≥8 indicates moderate neurological impairment after stroke (66). Most clinical trials set the NIHSS scores between 8 and 17, while there are some trials in which the NIHSS scores were set as low as 1 (67) or 2 (68). These negligible abnormalities caused by stroke may not respond well to neuroprotection. For example, an analysis of 1,733 patients with ischemic stroke administered blood pressure-lowering treatment revealed that there was a significant trend toward a better effect on functional outcome in patients with larger infarcts than in patients with smaller infarcts (lacunar infarction) (69). Moreover, we should acknowledge that the NIHSS score would be higher when small infarctions are located at the most densely populated motor fibers, which would result in poorer neuroprotective effects. When cerebral infarction occurs in the occipital lobe or the temporal lobe or some other areas that have fewer densely populated motor fibers, the NIHSS scores might be lower because most NIHSS parameters are relative to movement but do not incorporate cognition and other effects, and these patients would be excluded due to their lower NIHSS scores. Thus, various neuroprotective effects should be evident in some of these trials but might be counteracted by the negative outcomes and may result in negative results of neuroprotection. Therefore, the use of the NIHSS score as a major inclusion criterion for patients can lead to great study defects and shortcomings, which should be improved in future trials. The NIHSS content should also be amended to better reflect the actual conditions of stroke. In clinical practice, the Trial of Org 10172 in Acute Stroke Treatment (TOAST) classification (e.g., large-artery atherosclerosis, cardioembolism, small-vessel occlusion, stroke of other determined etiology, and stroke of undetermined etiology) (70) was primarily used for acute ischemic stroke. The large-artery atherosclerosis and cardioembolism have similar features on depriving cerebral blood supply with animal stroke models except for lacking of high risk factors and vasculopathy, while no clinical trials have selected the TOAST classification as their inclusion criteria to date, and only a few trials mentioned this in their data but still lack analysis of data on the neuroprotective effects according to the TOAST classification, such as the ICTUS trial (71). In addition, imaging-based patient selection, such as MRI-based or multimodal CT-based techniques, could provide better quantitative data and good visualizations for ischemic lesion size and location (72). Therefore, more attention should be paid to patient selection within the time window by combining the TOAST classification and imaging-based selection to evaluate the lesion size and location and ischemia etiology in the future clinical trials.

Furthermore, most neuroprotective agents have been tested in middle cerebral artery occlusion models in which the occluded arteries were reopened to deliver the agents to the salvageable tissues. However, most occluded arteries were not reopened in the enrolled patients with acute ischemic stroke when trials were performed, which may result in less effective delivery of neuroprotective agents to salvageable brain tissues despite some of the agents would *via* the collateral circulation. Although recent advances in endovascular therapies resulted in higher rates of revascularization after acute ischemic stroke, the therapeutic effects were not improved enough compared with the revascularization rates; one of the most important reasons may be revascularization without neuroprotection. Therefore, some promising trials were designed to investigate neuroprotective effects combined with endovascular therapy (Table 1), and better outcomes have been shown in a pilot study (38). However, the temporal dynamics of time to recanalization is also crucially important for the outcomes of patients with acute ischemic stroke. Because the longer of the time to recanalization, the high risk of poorer outcomes of patients would have (73–76). Moreover, despite successful revascularization, attention should also be paid to the flow in cerebral microvessels. Because the cerebral ischemia reperfusion has been shown in multivariate analyses is a surrogate marker of clinical outcomes independent of recanalization (77). Although the direct evidence of incomplete microcirculatory reperfusion (IMR) is still missing both experimentally and in clinical imaging due to the technical limitations, clinical studies have shown that a state of IMR is observed in approximately one-quarter of patients with successful recanalization showed by the non-invasive tools like CT or MRI to assess vessel status and tissue reperfusion in patients with acute ischemic stroke (78). In addition, the no-reflow phenomenon of cerebral microvessels or IMR in animal stroke models was evidenced by the results that pericyte contraction impairs capillary reflow followed by complete recanalization of an occluded cerebral artery (79, 80). Therefore, exploring potential mechanisms that contribute to no-reflow phenomenon that heavily preclude the delivery of blood, oxygen, and neuroprotectants to the salvageable brain tissue, and development of therapeutic approaches aiming at reducing microvascular obstructions may improve outcomes of patients (78).

**Table 1:** Ongoing clinical trials of neuroprotective agents combined with endovascular therapies in patients with acute ischemic stroke.

Name	Sample size	Registration number	Study phase	Compounds	Endovascular therapy	Treatment window	Primary outcome	Estimated completion
SONIC	210	NCT02831088	Phase 2	Neu2000KWL		8 h	mRS at 5 days, 4 and 12 weeks	July 2017
	100	NCT02054429	Phase 1	Insulin		8 h	mRS at 90 days	December 2018

ANSTROKE	90	NCT01872884		Sevorane, remifentanil	Embolectomy	8 h	mRS at 90 days	October 2016

MAVARIC	30	NCT02912663	Phase 1	Verapamil and magnesium sulfate	Thrombectomy		Number of participants with no symptomatic intracranial hemorrhage within 48 h after treatment	January 2019

ESCAPE-NA1	1,120	NCT02930018	Phase 3	NA-1	Endovascular thrombectomy	12 h	mRS at 90 days	April 2020

SAVER-I	30	NCT02235558	Phase 1	Verapamil	Intraarterial thrombolysis		Intracranial hemorrhage within 24–48 h after treatment	Completed

FAMTAIS	96	NCT02956200	Phase 2	Fingolimod	Alteplase bridging with mechanical thrombectomy	6 h	Salvaged ischemic tissue index (%) within 7 days	December 2018

KETA	50	NCT02258204	Phase 1	Ketamine	Recombinant of tissue type plasminogen activator	4.5 h	Cerebral infarction growth on diffusion weighted magnetic resonance imaging between admission and day 1	February 2018

## Novel Strategies for Neuroprotection

Stroke treatment should be a comprehensive strategy that involves reopening the occluded artery, using neuroprotective agents, and recovering neurofunction. Currently, successful endovascular therapy within the therapeutic time windows is promising to ensure that neuroprotective agents are delivered to salvageable brain tissues to exert their protective roles after stroke. In addition, due to the complexity of the ischemic cascade, numerous molecular targets should be addressed together in conjunction to achieve better neuroprotection. Accordingly, neuroprotection could lead to positive results only in trials with reasonable and feasible designs.

### Neuroprotection Before and After Revascularization

A foremost protective strategy is the early recanalization of the occluded arteries to restore flow to the ischemic brain region, which could successfully deliver more blood, oxygen, and neuroprotectants to achieve prospective efficacy. However, not all patients are suitable to receive vascular patency treatment due to high risks of hemorrhage (9, 54). As suggested above, imaging-based selection could provide the exact infarct lesion size and locations and collateral circulation of patients (72). Moreover, despite investigations of the neuroprotectants of more than 1,000 promising agents in clinical trials for acute ischemic stroke, few agents have been studied in patients whose occluded arteries were re-opened. Although greater neuroprotective effects of these agents might exist, this neuroprotective strategy failed because these agents could not successfully enter the salvageable tissues. Therefore, we speculate that revascularization might be the first step for neuroprotective strategy.

However, the effectiveness of recanalization therapies diminishes greatly >7.3 h from stroke onset (76), and this narrow therapeutic time window is limited to 10% or fewer patients who may be suitable for recanalization therapy (81). Therefore, prehospital first aid and emergency nursing are also important for stroke treatment. The use of ambulance-based thrombolysis has been shown to result in increased thrombolysis rates without an increase in adverse events (82). In addition, as endovascular therapies could result in symptomatic intracranial hemorrhage (53), protective measures should also be taken before the vascular patency procedure, for example, in the ambulance, to reduce hemorrhagic transformation of patients. In one clinical trial, however, the prehospital initiation of magnesium sulfate (a neuroprotective agent) therapy within 2 h after the onset of stroke did not improve disability outcomes at 90 days (83). Accordingly, we anchor our hope on shortening the time from stroke onset to admission and endovascular therapy because the longer the duration of cerebral ischemia followed by reperfusion, the higher rate of hemorrhagic transformation (10). In conclusion, neuroprotection strategies should be incorporated into the entire process (overall protection) from the onset of symptoms to postpatency. Thus, we propose that future clinical trials should be designed with a more comprehensive strategy that combines overall protection with vascular patency treatment.

### Cocktail Therapy

The damage mechanisms of cerebral ischemia injury are quite complex and involve several key signaling cascades of damaged brain tissues at different time points. For example, excitotoxicity occurs within several minutes and peaks within several hours as the first molecular mechanism that damages the ischemic brain. This is followed by oxidative and nitrosative stress and inflammation that occurs within several hours and is maintained at a higher level for several days after ischemia. These damage mechanisms sometimes work synergistically after cerebral ischemia. Therefore, it is difficult to foresee the therapeutic efficacy of neuroprotective agents used alone because they cannot be sufficient to suppress brain damage that results from different parts of the ischemic cascade. Following these pathophysiologic features of stroke, we propose that future translational trials should target multiple key molecular and cellular events in a sequential manner to reduce ischemia injury (cocktail therapy) in the acute phase (2), because some of the cellular and molecular events, like the appropriate neuroinflammation, may be benefited for the restoration of injured brain in the late phase of stroke (2). Although the use of cocktail therapy has been suggested since 2007 (84), there are still no reports of the clinical efficacy of this type of strategy. Nevertheless, we recommend that preclinical trials should be first conducted in large animal stroke models, such as NHPs, by combining failed neuroprotective drugs (e.g., anti-excitotoxicity + anti-inflammatory + anti-oxidant) with safety and tolerance tests to select the optimal combination of neuroprotective agents, and then, clinical trials should be carried out. Therefore, this cocktail therapy of neuroprotection combined with vascular patency may yield prospective efficacy for patients with acute ischemic stroke.

### Recirculation

Despite the high rates of revascularization with endovascular therapy, the structural and functional alterations in the microvasculature might also result in the no-reflow phenomenon (85), as capillaries may be still crowded with entrapped erythrocytes, leukocytes, fibrin, and activated platelets after the successful reopening of the occluded cerebral arteries in stroke models of rodents and primates (86–88). In addition, pericyte contraction induced by oxidative-nitrosative stress has also been shown to impair capillary reflow after ischemia (79, 80), which could be rescued by anti-oxidative-nitrosative stress agents by restoring pericyte dysfunction and microvascular patency (89). Moreover, the capillary transit time heterogeneity is also considered to influence the flow of cerebral microvessels and its metabolism, which also could hinder oxygen diffusion into brain tissue (90). Accordingly, more attention should also be paid to the no-reflow phenomenon in clinical practice to solve this issue. Unfortunately, there are still no clinical methods (i.e., imaging or ultrasound test) that can detect the no-reflow phenomenon in patients with ischemic stroke. Regardless of the lack of relative strategies, potential brain injury caused by the no-reflow phenomenon should not be ignored in clinical practice. Following the important clues of pericytes in regulating microvascular reflow in stroke animals, the exact regulating mechanisms should be investigated in both animals and patients subjected to ischemic stroke. In addition, cerebral venous systems are part of the cerebral circulation and can respond to acute brain injury by regulating blood flow disharmony (18), which means that the unimpeded flow of the cerebral venous system is also important for the recirculation of cerebral vessels, not just reperfusion. Therefore, neuroprotective agents may now hold promise for promoting recovery and minimizing injury when used in conjunction with recirculation strategies after stroke and should be extensively explored in future investigations and clinical trials.

### Collateral Circulation

Intriguingly, good pial or leptomeningeal collateral circulation has been shown to extend the time window for endovascular procedures (91). For example, 40% of patients who were reperfused at later time points still achieved independent functional outcomes from endovascular therapy (92), which indicates the important role of collaterals in predicting favorable outcomes (93, 94). This role of collaterals was also confirmed by a subgroup analysis of the IMS III trial, which showed that a more robust collateral grade was associated with better clinical outcomes (95). Good collateral circulation could significantly reduce the risk of symptomatic intracranial hemorrhage and improve early neurological improvement after thrombolytic and endovascular therapies (94, 96). Moreover, good pretreatment collateral circulation has been shown to be associated with superior functional outcomes at 3 and 6 months (94). These results showed that good collateral circulation can enhance the tolerance of stroke. Accordingly, increasing the effective collateral circulation in humans who suffer from high risk factors for stroke may enhance the protection against brain injury induced by stroke and could also provide more vascular channels for the delivery of neuroprotective agents to salvageable brain tissues after stroke.

Collateral-enhancing strategies are important ways to restore blood flow within ischemic regions (72, 97). Strategies that aim to manipulate the hemodynamics to increase brain–blood flow include induced hypertension (97), lying flat head position (98, 99), and volume expansion (100). Other strategies, such as hyperoxia (101), remote ischemic preconditioning (102), and physiological ischemic training (103), have also been shown to increase cerebral blood flow (CBF) in ischemic regions. Although these strategies that might impact collateral circulation have been investigated in preclinical and/or clinical studies of ischemic stroke, none have been applied to clinical practice. Therefore, the road to improving CBF in ischemic regions remains challenging.

### Angiogenesis

On the other hand, enhancing angiogenesis (capillary formation from pre-existing vessels) and vasculogenesis (*de novo* capillary formation) to re-establish collateral circulation might also benefit patients who have high stroke risk factors. Treatment with various pharmacological therapies, including the phosphodiesterase type 5 inhibitor sildenafil (104, 105), vascular endothelial growth factor-A (106, 107), has been shown to promote angiogenesis and vasculogenesis. Large randomized trials in patients with acute stroke showed negative results (65, 108), and such failures may be due to inadequate patient selection and a lack of assessment of the effects of such interventions on collateral blood vessels and collateral flow (72). Furthermore, more attention should be paid to the complication of increasing collateral circulation because the augmented collateral circulation may result in hemorrhagic transformation (109). Therefore, re-establishing effective and functional collateral circulation while not just promoting neovascularization is crucial for the prevention of these people and supports the use of preventive strategies, such as antiplatelet drugs, to enhance functional collateral circulation. Accordingly, the second major question is how to promote the growth of new vessels into functional microvessels that are non-fragile and are influenced by blood pressure.

### Hemorrhagic Transformation After Revascularization

As one of the most serious complications of revascularization, better intervention strategies for hemorrhagic transformation are still currently lacking (10, 110). Hemorrhagic transformation occurs in 10–40% of patients with ischemic stroke who undergo thrombolytic or endovascular therapy (10, 111) and contributes to the increase in stroke morbidity and mortality (112). It must be questioned why the hemorrhagic transformation occurs when cerebral blood is restored, while it does not occur in other ischemic tissues, such as ischemic heart, liver, and kidney. One of the major reasons is that the vascular components (e.g., pericytes, endothelial cells and smooth muscle cells) and their biofunctions differ between cerebral and periphery vasculature. Therefore, damaged vasculature should be the principal culprit that causes hemorrhagic transformation when CBF is restored. The abnormally permeable blood–brain barrier that results from ischemia of the capillary endothelium allows the extravasation of blood (110). Many factors, including reactive oxygen species (113), leukocytes (114), affect BBB permeability and have been evaluated in animal models. Although many compounds that target these factors have been shown to decrease hemorrhagic transformation in animals (115), to date, none of them have been successfully translated. For instance, NXY-059 showed promise in reducing hemorrhagic transformation in a rabbit stroke model (116), but it failed to reduce hemorrhagic transformation in patients with stroke (27).

The reasons why promising therapies for hemorrhagic transformation of stroke have failed in clinical translation studies remains unclear. It highlights that damage to the BBB caused by multiple mechanisms during ischemia/reperfusion injury may not be sufficiently protected by pharmacological drugs alone in human hemorrhagic transformation. Based on revascularization in both animals and humans with stroke, the use of multiple protective drugs may be necessary to evaluate the prevention of hemorrhagic transformation. In addition, more effort should be focused on the exploration of unknown BBB damage mechanisms that may involve the regulatory role of pericytes, the transcytosis of endothelial cells, etc. An improved understanding of hemorrhagic transformation is essential to reduce its impact on patients with ischemic stroke and to improve our management ability to restore blood flow to the ischemic brain without inducing this complication (10).

## Conclusion

Although the road toward achieving neuroprotection is extremely challenging, the higher rate of revascularization of occluded arteries acquired by endovascular therapies provides more ability to deliver neuroprotectants to salvageable brain tissues after ischemic stroke. As multiple molecular events involved in the ischemic stroke induce brain damage, a single target of neuroprotection will not provide the expected therapeutic effects. Therefore, multitarget neuroprotectants (cocktail therapy) immediately administered to patients after successful revascularization may be promising for conquering these issues. In addition, more attention should be paid to enhance collateral circulation and prevent the no-reflow phenomena and hemorrhagic transformation after cerebral ischemia. We hope to address the following important strategies to provide better comprehensive treatment of acute ischemic stroke: prehospital first aid and emergency nursing, endovascular therapy combined with cocktail neuroprotectants usage, and the prevention of hemorrhagic transformation. A perspective that considers a comprehensive strategy is warranted and may speed the progress toward improving the neuroprotection of stroke.

## Author Contributions

X-YX and Q-WY conceived the idea and wrote the first draft of the manuscript. LL conceived the content related to preclinical data. All authors approved the final version of the manuscript.

## Conflict of Interest Statement

The authors declare that the research was conducted in the absence of any commercial or financial relationships that could be construed as a potential conflict of interest.
